# Electrically-Driven Zoom Metalens Based on Dynamically Controlling the Phase of Barium Titanate (BTO) Column Antennas

**DOI:** 10.3390/nano11030729

**Published:** 2021-03-14

**Authors:** Ning Xu, Yuan Hao, Kaiqian Jie, Shuai Qin, Hui Huang, Li Chen, Hongzhan Liu, Jianping Guo, Hongyun Meng, Faqiang Wang, Xiangbo Yang, Zhongchao Wei

**Affiliations:** Guangdong Provincial Key Laboratory of Nanophotonic Functional Materials and Devices, School of Information and Optoelectronic Science and Engineering, South China Normal University, Guangzhou 510006, China; xuning199405@outlook.com (N.X.); primulaaaaaaa@gmail.com (Y.H.); kqjie@m.scnu.edu.cn (K.J.); shuaiqin@m.scnu.edu.cn (S.Q.); huihuang@m.scnu.edu.cn (H.H.); lichen@m.scnu.edu.cn (L.C.); lhzscnu@163.com (H.L.); guojpgz@163.com (J.G.); hymeng@scnu.edu.cn (H.M.); fqwang@scnu.edu.cn (F.W.); xbyang@scnu.edu.cn (X.Y.)

**Keywords:** metalens, barium titanate, electro-optic, zoom

## Abstract

The zoom metalens has been a research hotspot for metasurfaces in recent years. There are currently a variety of zoom methods, including dual metalenses, micro-electromechanical system metalenses, polydimethylsiloxane metalenses and Alvarez metalenses. However, for most metalenses, zooming is achieved by manipulating the relative displacement of two or more metasurfaces. Therefore, these methods seem inadequate when faced with more precise zooming requirements, and the precise control of the phase distribution cannot be achieved. In this paper, we innovatively propose an electrically-driven zoom metalens (EZM) of one-dimensional based on dynamically controlling barium titanate (BaTiO_3_, BTO) antennas. Using the electro-optic effect of BTO crystals, we can apply a voltage to change the refractive index of BTO nanopillars (*n* = 2.4–3.6), thereby accurately controlling the phase distribution of column antennas. The proposed EZM can achieve 5× zoom (*f* = 10–50 μm), with advantages, such as high-speed optical amplitude modulation, ultra-compactness, flexibility and replicability. It can be applied in fields that require ultra-compact beam focusing, zoom imaging, and microscopic measuring.

## 1. Introduction

A zoom lens system is one of the most important optical systems whose applications can be found in various imaging systems. Artificial composites with equivalent constitutive parameters synthesized by the subwavelength structure are called metamaterials [1], which have strong electromagnetic manipulation ability. The concept of metamaterials and metasurfaces based on sub-wavelength makes the research of micro-flat lens get further development, and a variety of metasurface devices are designed according to the surface plasmon resonance theory [2]. In recent years, with the continuous deepening of research on dynamic control of metasurfaces, the research of zoom metalenses has become one of the hot topics in the field of metalenses. Several zoom metalens methods have been achieved [3,4,5]. Some researchers combine the zooming method in traditional optics with metalens. For example, Ehsan Arbabi et al. demonstrated tunable metasurface doublets based on micro-electromechanical systems [6,7], Nazmi Yilmaz et al. proposed highly efficient rotationally tunable metasurface lens structures inspired by Moiré lenses [8,9] and Shane Colburn et al. built a 1 cm aperture varifocal metalens system at 1550 nm wavelength inspired by an Alvarez lens [10]. Some researchers used unique optical modulation methods in the metasurfaces to realize the step zoom lens. For example, Guoxiang Zheng et al. demonstrate a dual field-of-view step-zoom metalens [11]. Someone uses special materials to achieve zoom. For example, Shiqiang Li etc. proposed a concept of tunable dielectric metasurfaces modulated by liquid crystal [12,13]; Weiming Zhu et al. used liquid metal to achieve a tunable flat lens [14]. In addition, there are also some special zoom methods that are achieved by using flexible, stretchable substrate polydimethylsiloxane [15,16] or graphene [17,18,19]. However, for most metalenses, zooming is realized by manipulating the entire metasurfaces, such as translation, rotation and stretching. Therefore, these methods seem inadequate whEn faced with more precise zooming requirements, and the precise control of the phase distribution cannot be achieved.

**Figure 1:**
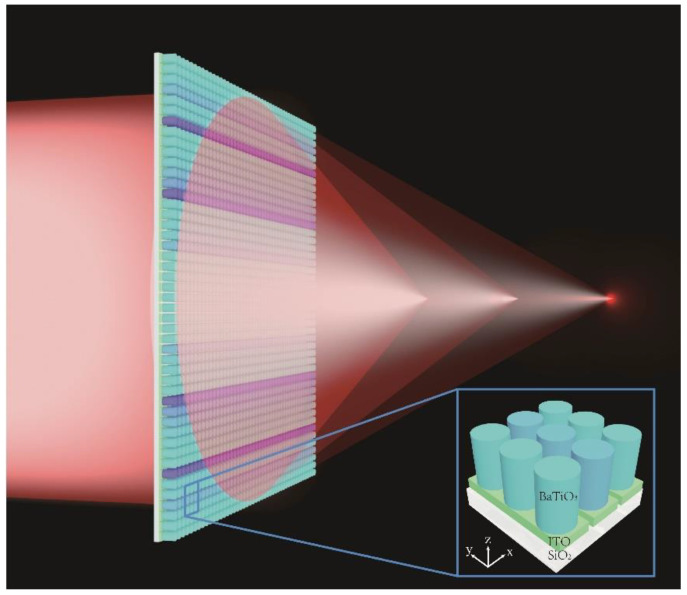
Schematic of electrically driven zoom metalens (EZM) (1D) in visible light. The illustration shows the arrangement of the barium titanate (BTO) antennas that we only change the voltages of antennas instead of the radius.

In this paper, an electrically-driven zoom metalens (EZM) is proposed based on dynamically controlled barium titanate (BTO) antennas in visible light (Figure 1.) based on the commercial software FDTD Solutions (Lumerical Inc.). Due to the electro-optic (EO) effect of BTO crystals [20], we can apply a voltage to change the refractive index of BTO nanopillars (*n* = 2.4–3.6). The working method and schematic are shown in Figure 1. Different from previous research, instead of manipulating the entire metasurface, we propose a new method of controlling the change of the refractive index of BTO antennas by applying external voltages to achieve a phase change. The modulation area is more precise, and the controlling of phase is more flexible. In addition, we do not need to change the geometry of the nanopillars. The phase distribution of antennas can be accurately controlled to achieve a phase coverage of 0–2π. By controlling the voltage (*V* = 0–63 V), the proposed EZM can achieve a wide range of focal length changes (5×, *f* = 10–50 μm). When the focal length changes in the range of 10–35 μm, the maximum of FWHM is 0.56 μm, which can achieve near-diffraction limit focusing. Based on this method of independent controlling of each antenna, the EZM has many advantages, such as high-speed optical amplitude modulation, ultra-compactness, flexibility and replicability. Our method can be applied in fields that require ultra-compact zoom imaging, microscopic imaging and beam focusing.

## 2. Materials and Methods

**Figure 2:**
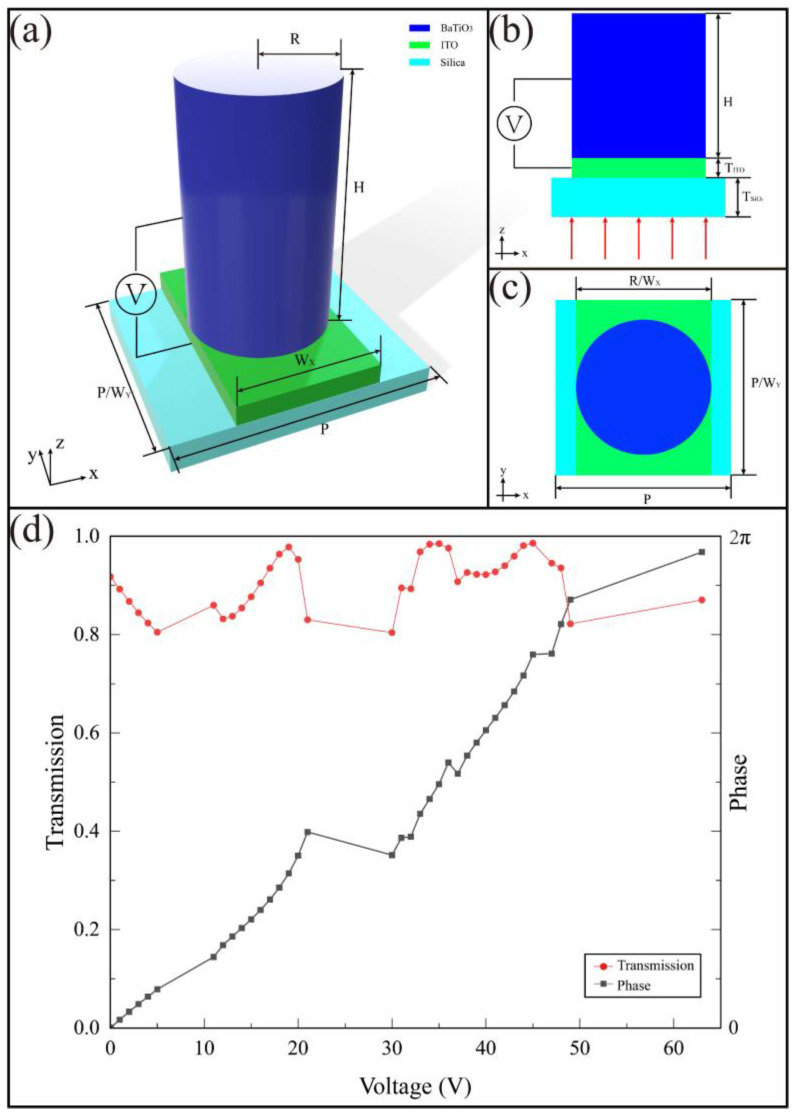
(**a**–**c**) Diagrams of the meta-atom. Along with the longitude, the materials are BTO (*R* = 0.17 μm, *H* = 0.5 μm), ITO (*T_ITO_* = 0.1 μm, *W_x_* = 0.34 μm), silica (*T_Silica_* = 1 μm), and the lattice constant *P* = 0.4 μm; (**d**) the transmission (red line) and phase change (black line) of meta-atom when the applied voltage of BTO changes from 0 V to 63 V by commercial software FDTD Solutions (Lumerical Inc.).

Figure 2a illustrates the meta-atoms of the designed EZM whose work wavelength is 0.6 μm. From top to bottom are BTO antenna, indium tin oxides (ITO) transparent electrode and glass substrate. In the calculation of the FDTD solutions, we use symmetric and antisymmetric boundary conditions to scan the sub-elements to find the optimal element size. The unit size of the meta-atom is optimized by parameter sweeps, which are obtained as *H* = 0.5 μm, *T_ITO_* = 0.1 μm, *T_Silica_* = 0.2 μm, *R* = 0.17 μm. The lattice constant P is chosen to be 0.4 μm to reduce the near-field coupling of adjacent waveguides. The ITO layer is used as a transparent electrode to connect an external voltage to the BTO antenna, and its length in the x-axis (*W_x_*) is 0.34 μm.

The phase realization mechanism is described by the waveguide model. Here, we introduced a single step-index circular waveguide model and calculated the phase imparted solely by the waveguiding effect, which is given by [21]:

(1) $$\varphi_{WG}=\beta H=\frac{2\pi }{\lambda_{d}}n_{eff}H,$$

where *n_eff_* is the effective index of the fundamental mode (HE_11_), and *H* (nanopillars height) is the propagation length. The *n_eff_* can be readily computed by a single step-index circular waveguide model. In the FDTD solution, we calculate the transmission and phase of the antenna under an applied voltage of 0–100 V and establish a data set based on it. There is a resonance phenomenon between the BTO and SiO_2_, which is a consequence of the thicknesses of the layers in the stack BTO-ITO-SiO_2_. Thereby, we select 39 external voltage values that are conducive to the design of the metalens in the data set, all of which are concentrated between 0–63 V. Figure 2d shows the transmission (red line) and phase gradient (black line) under different applied voltages (0–63 V) by commercial software FDTD Solutions (Lumerical Inc., Vancouver, BC, Canada).

In this paper, the linear electro-optic effect is utilized, where changes in the refractive index are proportional to an electric field, called the Pockels effect. The BTO has high nonlinear optical and EO properties, including negative birefringence, two refractive indices, two ordinary optical axes that are set along the x-axis and y-axis, and an extraordinary optical axis along the z-axis in the following [22]. The high-quality, single-crystalline BTO can be obtained using a combination of epitaxy and direct wafer bonding. The approach yields dense, crystalline and tetragonal BTO films, which are needed to preserve the Pockels effect [18]. Next, the BTO film is prepared into nanopillar antennas by ion beam etching technology. The application of an electric field on the BTO will increase the refractive index of antennas, and thus the phase distribution of meta-atoms can be tuned. The ordinary refractive index of BTO can be written as [23]:

(2) $$n=n_{0}+\frac{1}{2}n_{0}^{3}r_{51}E,$$

where *n_0_* = 2.4 is the ordinary refractive index of BTO under no electric field, *r_51_* = 1300 pm/V is the EO coefficient. $E=\frac{V}{d}$, where *E* is the external electric field strength, *V* is the external voltage, and *d* is the thickness of the EO layer, which equals the height H of BTO nanocolumns. Although the extraordinary refractive index also varies with the applied voltage, we set the extraordinary axis in parallel to the propagation direction, and thus it does not influence the propagation of the waves, so that only an ordinary index needs to be considered [24].

**Figure 3:**
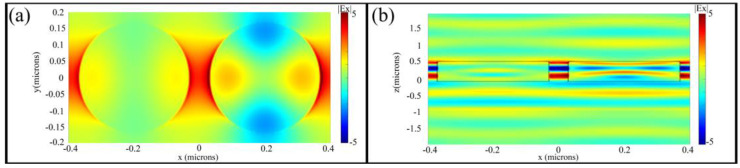
(**a**,**b**) the electric near field real(Ex) distribution in xy-plane and xz-plane by commercial software FDTD Solutions (Lumerical Inc.). The applied voltages of the left and right antennas are 15 V and 35 V, respectively.

The electric near field *real(Ex)* distribution in xy-plane and xz-plane are illustrated in Figure 3a,b based on the commercial software FDTD Solutions (Lumerical Inc.). The left and right pillars applied voltages of 15 V and 35 V, respectively. Our work differs from previous research in that we do not need to change the geometry of the nanopillars but control the change in refractive index by applying an external voltage to achieve a phase change.

## 3. Results and Discussion

**Figure 4:**
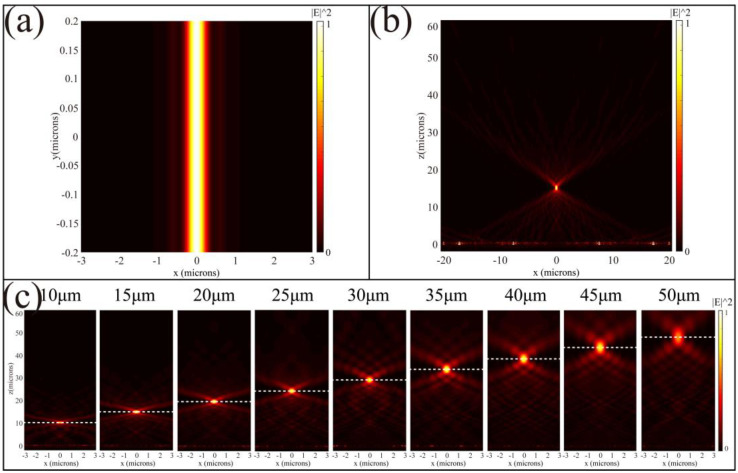
Normalized intensity distribution of simulated focusing results based on the commercial software FDTD Solutions (Lumerical Inc.). (**a**,**b**) The normalized intensity distribution of the electric field of the focal and the transmission planes when the design focal length is 15 μm. (**c**) EZM zoom at focal lengths of 10–50 μm.

Based on BTO electronically controlled nanopillars, a one-dimensional zoom metalens EZM is designed. The components of the metalens are the BTO nanopillars and ITO transparent electrodes on the glass substrate (SiO_2_). In order to achieve focus, each nanopillar in the position must meet the requirement of phases below [25]:

(3) $$\varphi \left(x\right)=2\pi n-\frac{2\pi }{\lambda_{0}}\left(\sqrt{x^{2}+f^{2}}-f\right),$$

where *x* denotes the center coordinates of the unit located in the x-axis, *f* is the focal length, $\lambda_{0}$ is the operation wavelength of 0.6 μm, and *n* is an arbitrary integer. In this paper, the length of EZM in the *x*-axis is 40 μm, and the length of EZM in the y-axis is semi-infinite, which is designed as a periodic boundary condition. Based on the commercial software FDTD Solutions (Lumerical Inc.), Figure 4a,b shows the normalized intensity distribution of the electric field of the focal and transmission planes when the designed focal length is 15 μm, respectively. Since the designed metalens is one-dimensional, the focus pattern of the focal plane is a focal line. Next, we change the applied voltage of the subunit antenna and the focal length from 10 μm to 50 μm in 5 μm steps. The normalized intensity distribution of the electric field of the transmission plane with different focal lengths is shown in Figure 4c. The focusing effect gradually decreases with increasing focal length, which is affected by changes in the numerical aperture (NA).

**Figure 5:**
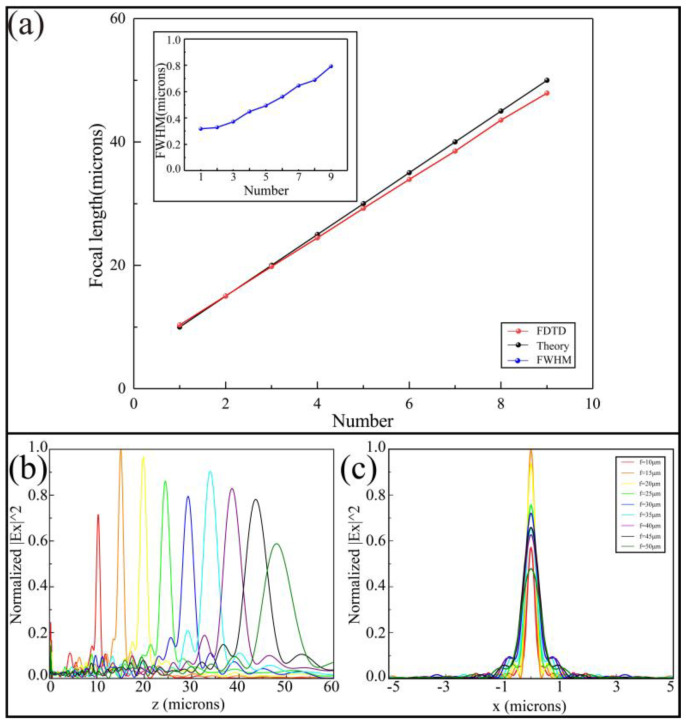
Brief evaluation of focusing quality. (**a**) The degree of focal length shift. The illustration depicts the change of half-height width at different focal lengths. (**b**,**c**) The normalized electric field intensity distribution of different focal lengths in transmission plane and focal plane, respectively.

According to the formula $NA=D/x\sqrt{f^{2}+D^{2}/4}$ [26], when the *D* is fixed, the increase in focal length will lead to the reduction of the NA, which results in a decrease in resolution and an increase in depth of field. In order to evaluate the imaging quality of EZM briefly, we analyzed the focus shift, intensity distribution and full width at half maximum (FWHM) at different focal lengths. We select nine focal length values of 10–50 μm, which are called numbers 1–9. The degree of focal length shift is shown in the main image of Figure 5a. The red and black lines represent the designed focal length and the focal length simulated in FDTD, respectively. As the distance between the focal length and the design value (15 μm) increases, the deviation of the focal length from the theoretical value also increases. The illustration of Figure 5a shows the change of FWHM at different focal lengths, which is depicted by the blue line. It can be seen from the results that as the focal length increases, the values of FWHM also increase so that the focusing result worsens. When the focal length changes in the range of 10–35 μm, the maximum of FWHM is 0.56 μm, which can achieve near-diffraction limit focusing. A similar conclusion can be obtained from Figure 5b,c, which indicates the normalized electric field intensity distribution of different focal lengths in the transmission plane and focal plane, respectively. The color and corresponding focal lengths in Figure 5 are shown in Table 1 below.

In this paper, the initial focal length is designed to be 15 μm. As the focal length deviates from the design value, the degree of phase matching of the metalens array will also decrease. In addition, the metasurface has certain restrictions on light transmission. The larger the focal length, the lower the light transmission at the focus. (The transmissions at focus are 41% and 25% when *f* = 15 and 50, respectively.

## 4. Conclusions

In conclusion, in this paper, we innovatively proposed an electrically driven zoom metalens (EZM), which is based on dynamically controlled barium titanate (BTO) antennas at the visible work wavelength. Using the EO effect of the BTO crystal, we can apply a voltage to change the refractive index of BTO nanopillars. Different from previous research, instead of manipulating the entire metasurface, we propose a new method of controlling the change of the refractive index of BTO antennas by applying external voltages to achieve a phase change. The modulation area is more precise, and the controlling of phase is more flexible. In addition, we do not need to change the geometry of the nanopillars. Under the control of voltage (*V* = 0–63 V), the proposed EZM can achieve a wide range of focal length changes (5×, *f* = 10–50 μm). When the focal length changes in the range of 10–35 μm, the maximum of FWHM is 0.56 μm, which can achieve near-diffraction limit focusing. Based on this method of Independent controlling of each antenna, the EZM has many advantages, such as high-speed optical amplitude modulation, ultra-compactness, flexibility and replicability, which can be widely utilized in fields that require ultra-compact zoom imaging, microscopic imaging and beam focusing.

## Figures and Tables

**Table 1:** Table of the color and corresponding focal lengths.

Number	Color	Focal Length
1	Red	10 μm
2	Orange	15 μm
3	Yellow	20 μm
4	Green	25 μm
5	Bule	30 μm
6	Lake Blue	35 μm
7	Purple	40 μm
8	Black	45 μm
9	Olive	50 μm

## Data Availability

No new data were created or analyzed in this study. Data sharing is not applicable to this article.
